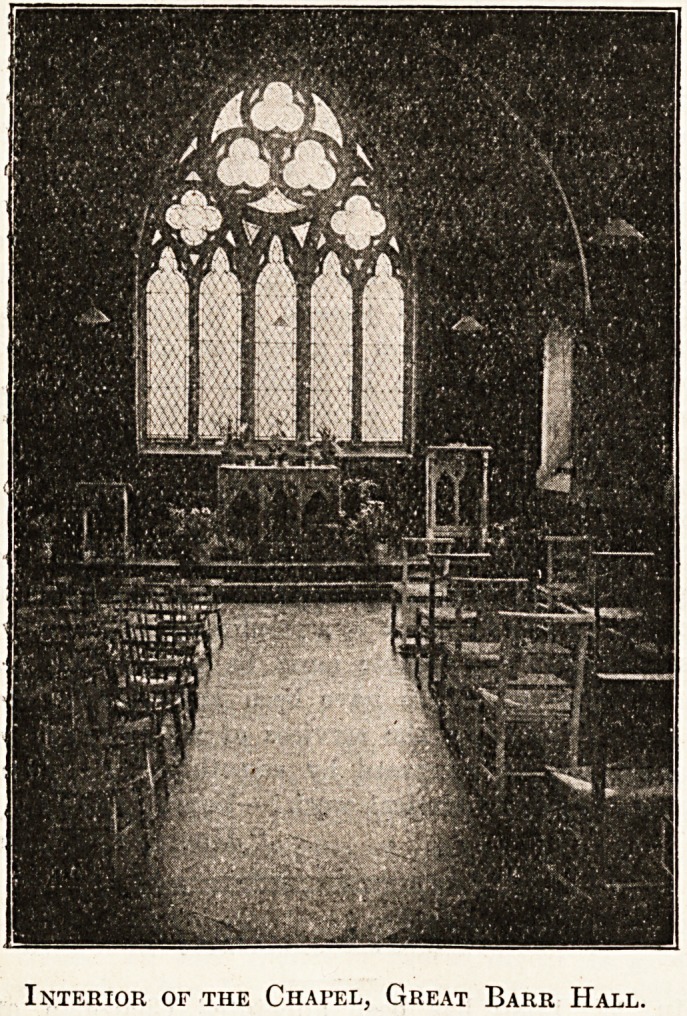# Chapel of Great Barr Hall Institution, near Birmingham

**Published:** 1915-05-01

**Authors:** 


### REMARKABLE HOSPITAL CHAPELS.

(Contributions Invited.)

### Chapel of Great Barr Hall Institution, near Birmingham.

LIKE so many things connected with the beautiful Home for Children at Great Barr Hall, the chapel is certainly unique. The furniture is all plain and substantial. On one side are chairs for the staff and on the other side

THE CHAPEL, GREAT BARR HALL.

rows and rows of baby-chairs for the children. All those who are past the infant stage are allowed to go to chapel. Their ages range from two to five years. Very beautiful, indeed, is it to hear their baby voices singing

INTERIOR OF THE CHAPEL, GREAT BARR HALL.

hymn after hymn and joining in the Lord's Prayer. Occasionally there is a christening.

The chapel is always kept well supplied with plants and flowers from the hot-houses and gardens of the Home. The altar curtains, which are the only curtains in the institution, are of rich velvet with Oriental border.

The building was designed by the famous architect, Sir Gilbert Scott. The Rev. A. F. Giddings is chaplain.

### LADY SCOTT'S EPITAPHS ON ANIMALS.

The hall formerly belonged to Lady Bateman Scott, who was a great lover of animals. Outside the chapel are buried several of her pets. Their graves are covered with flat slabs. The following are the inscriptions :

Farewell, dear Billy, never more
Thy cheery bark I hear.
O'er thy little grave I pour
My grief with many a tear.
And yet thou'rt happy in thy rest,
And from all troubles free;
Thy long bright life was surely blessed,
So good thou wert to me.

Died April 24, 1886. M. A. B. S.

Good-bye, dear Spot,
They say thou art for ever gone
And ne'er canst be again.
But who can tell what He may do
Who made both dogs and men?
I would not venture to pronounce
What may be His decree,
But He Who all things can restore
May have a life for thee.

Died March 18, 1892. M. A. B. S.

In Memory of my dear old Parrot, who died November 24, 1895. A friend and companion of over twenty-two years.—M. A. BATEMAN SCOTT.

My dear cat Tommy, who left this world January 8, 1901.—M. A. BATEMAN SCOTT.

These are unusual enough in a hospital chapel to be recorded.

## Figures and Tables

**Figure f1:**
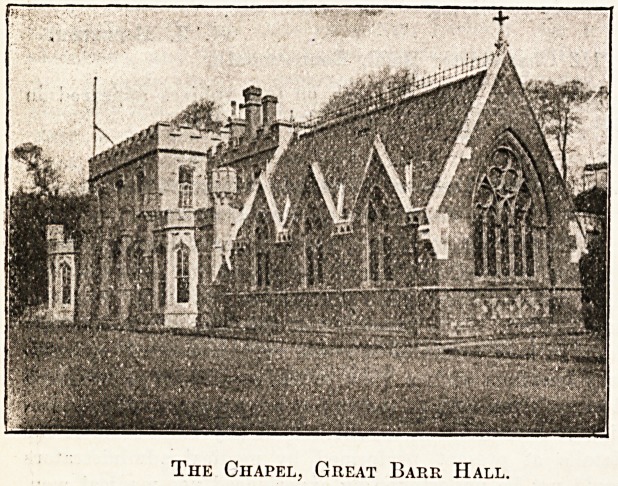


**Figure f2:**